# Supplementary material for: Fast and compact matching statistics analytics
Source: Bioinformatics. 2022 Feb 4;38(7):1838–45. doi: 10.1093/bioinformatics/btac064 (PMC9665870; doi:10.1093/bioinformatics/btac064)
Supplement: btac064_Supplementary_Data [file btac064_supplementary_data.pdf]

# Supplementary material for: "Fast and compact matching statistics analytics"

Fabio Cunial Olgert Denas Djamal Belazzougui

## 1 Details on the experiments

We run our parallelism experiments on a server with four Intel Xeon E7-4830v3 processors, each with 12 cores, a base frequency of 2.1 GHz, and a maximum frequency of 2.7 GHz. The machine has one terabyte of RAM and no other user. We compile our code with `gcc 6.2.0`.

Since our parallel algorithm might not scale well when the query is very similar to the text, we experiment with the following pairs of similar genomes and proteomes: *Arabidopsis lyrata* and *Arabidopsis thaliana*; *Zea mays* and *Oryza sativa*; *Rattus norvegicus* and *Mus musculus*; *Gallus gallus* and *Taeniopygia guttata*; *Brugia malayi* and *Caenorhabditis elegans*; *Drosophila melanogaster* and *Anopheles gambiae*; *Pan troglodytes* and *Homo sapiens*. When building each dataset, we download the latest assembly from NCBI and we concatenate its sequences using a separator that is not in the alphabet, replacing runs of undetermined characters with a single occurrence of the separator. To probe the high-similarity regime, we compare 28 random pairs of human chromosome 1 taken from the 1000 Genomes Project[1]. We transform the set of variants from each individual into two full copies of chromosome 1, of length approximately 215 million each, using the `vcf2multialign` tool[2].

We run our sequential range query experiments on a server with two Intel Xeon E5-1650v4 processors, each with 6 cores, a base frequency of 3.6 GHz, and a maximum frequency of 4 GHz. The machine has 128 GB of RAM and no other user. We compile our code with `gcc 8.4.0`.

In some experiments we need to approximate a use case in which the genomes or proteomes of different species are compared to one another: for this we use a dataset of 24 eukaryotic species with relatively large genomes that has been used for whole-genome phylogeny reconstruction by average common substring before [8]. Such genome files range from approximately 87 million to 5.8 billion characters. We call this the *ACS dataset* in what follows.

[1] `ftp://ftp.1000genomes.ebi.ac.uk/vol1/ftp/release/20130502/ALL.chr1.phase3_shapeit2_mvncall_integrated_v5a.20130502.genotypes.vcf.gz`

[2] `https://github.com/tsnorri/vcf2multialign`

## 2 Compressing frequency and position arrays

Given a position $i$ of the query $S$, knowing the frequency of the matching statistics string $S[i..i + \mathsf{MS}_{S,T}[i] - 1]$ in the text $T$ can be useful in genome-genome and read-genome comparison, since it can tell for example whether the longest match belongs to an exact repeat of $T$ (see e.g. Figure 22 in the supplement). One might also want to keep the exact values of MS just for the positions with low frequency, i.e. one might want to compute a *frequency thresholded* MS array in which every window of `ms` between two low-frequency one-bits can be permuted to achieve compression. We define the *frequency array* $\mathsf{F}_{S,T}[0..m-1]$ to be such that $\mathsf{F}_{S,T}[i] = f_T(S[i..i + \mathsf{MS}_{S,T}[i] - 1])$.

The algorithm for computing `ms` described in Section 2.2 can be easily adapted to compute the $\mathsf{F}$ array as well. Specifically, during the first scan of $S$ (from right to left), we set $\texttt{runs}[i] = 0$ iff either: (1) $\mathsf{MS}[i] \neq \mathsf{MS}[i-1]-1$, as before, or (2) if $\mathsf{MS}[i] = \mathsf{MS}[i-1]-1$ but $\mathsf{F}[i] \neq \mathsf{F}[i-1]$. We can detect the latter case since we know the frequency of the current string after every backward step. Consider now the first few operations of the second scan of $S$ (from left to right): we managed to match some prefix of $S$, and we are now witnessing a Weiner link fail from some node $v$ of $\overline{\mathsf{ST}}$, thus we move to the parent $u$ of $v$. Node $u$ must have a different frequency in $T$ than $v$, so we know that the first parent operation we take leads to the position $i$ of the first zero-bit from the left in the `runs` bitvector. We can measure $f(u)$ with the topology, and we know that $\mathsf{F}[j] = f(v)$ for all $j \in [0..i-1]$. We repeat this process after every parent operation. At some point the Weiner link succeeds, so we derive the updated frequency from the BWT and we restart the whole process. This algorithm can be parallelized using the same methods as in Section 3.

When $S$ and $T$ are similar, most matches are long and the values of $\mathsf{F}$ are more likely to be small or equal to one (and vice versa when $S$ and $T$ are dissimilar), thus delta-coding $\mathsf{F}$ achieves better compression when $S$ and $T$ are similar. Moreover, assume that most edges of $\overline{\mathsf{ST}}_T$ are labelled by long strings. If $S$ and $T$ are dissimilar, they mostly have short matches, the loci of such short matches have low tree depth in $\overline{\mathsf{ST}}_T$, and they do not create long runs in $\mathsf{F}$. If $S$ and $T$ are similar, they have many long matches, the loci of such long matches are more likely to have large tree depth in $\overline{\mathsf{ST}}$, and this can induce long runs in $\mathsf{F}$. If the edges of $\overline{\mathsf{ST}}$ have short labels, there is little to be gained in having $S$ similar to $T$. In practice, for pairs of genomes from different species, representing $\mathsf{F}$ as a delta coding of its runs produces bigger files than just delta-coding every entry of $\mathsf{F}$ in isolation; but for pairs of human individuals, the run-length encoded $\mathsf{F}$ is about 15 times smaller (data not shown for brevity). Note that the values in the run-length encoded $\mathsf{F}$ can be accessed easily using the `runs` bitvector.

We conclude this section by mentioning one more array that is easy to compress. Let $\mathsf{P}_{S,T}[1..|S|]$ be the array that stores at position $i$ an arbitrary location of $T$ at which $S[i..i + \mathsf{MS}[i] - 1]$ starts[3] [3]. Rather than storing all locations, it suffices to store just

[3] One could of course define lossy variants in which the locations of short matches are discarded.

those of the *informative positions*, i.e. of positions $i$ such that $\mathsf{MS}[i] > \mathsf{MS}[i-1]-1$: for every other position $j$, $\mathsf{P}[j]$ can be reconstructed by recurring on $\mathsf{P}[j-1]+1$. Statistical properties of informative positions in random sequences were described by [5]. In practice, when comparing genomes from different species, approximately half of all positions are informative; this fraction ranges from 0.7 to 0.2 in proteomes, whereas in human individuals it becomes smaller than 0.01 (see Figure 5 in the supplement). Note also that, in the range-max queries described in Section 5 of the main text, the maximum of each block is assumed by an informative position, thus it suffices to consider just those during construction.

One can imagine other position arrays that might be useful in genome comparison, for example an array that stores zero if a match occurs in distinct chromosomes of the text, or the identifier of the only chromosome that contains the match otherwise.

## 3 Acknowledgements

We thank Giorgio Vinciguerra for help with the software in [2], and Massimiliano Rossi and Dominik Koeppl for help with the software in [3].

## 4 Supplemental figures

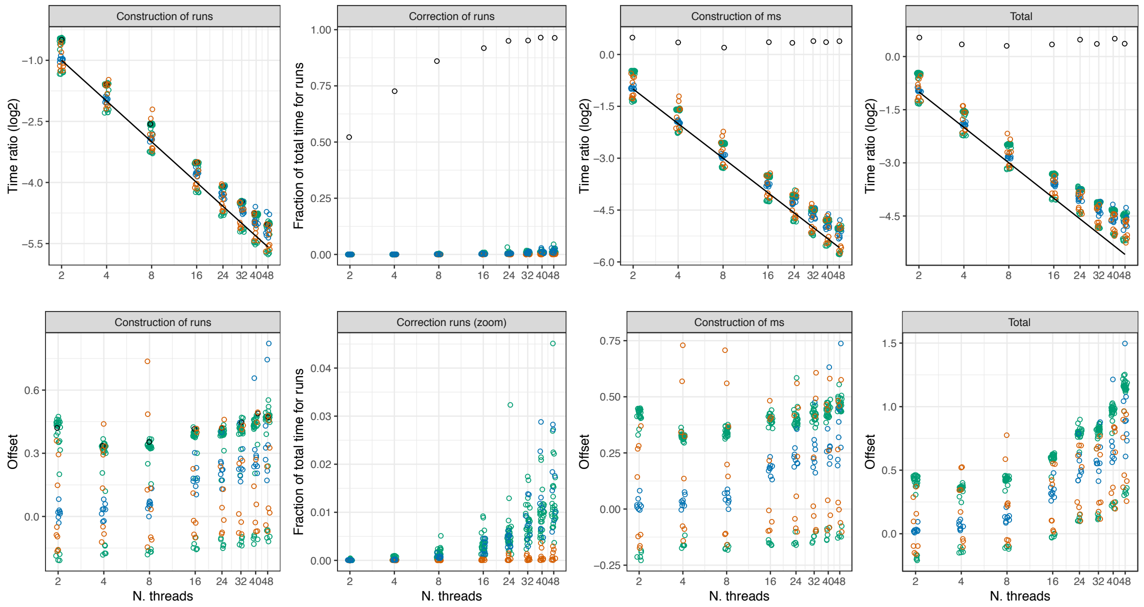


Figure 1: Running time of the parallel implementation as the number of threads increases. Red: genomes of similar species; blue: proteomes of similar species; green: pairs of human chromosome 1 from distinct random individuals; black circles: identical query and text (human chromosome 1 from two random individuals); black line: $1/t$, where $t$ is the number of threads. Time ratio: time of the parallel implementation divided by the time of the sequential implementation. Offset: $(r - 1/t)/(1/t)$, where $r$ is the time ratio and $t$ is the number of threads. Correction of `ms` is not shown since it is negligible in all cases.

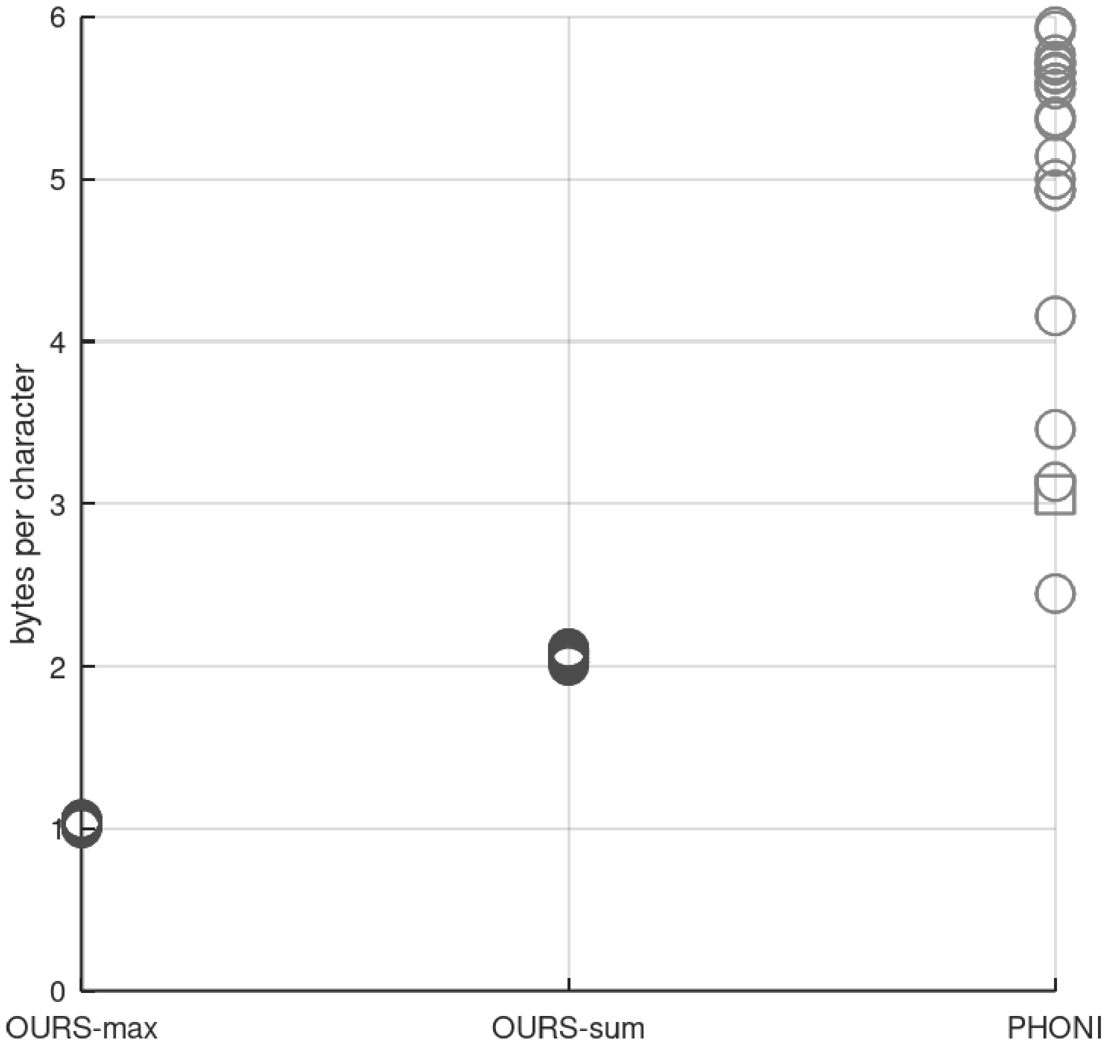


Figure 2: Comparing the size of the indexes used by our implementation ("OURS", [1]) to those used by the implementation in [3] ("PHONI"), on all genomes in the ACS dataset. Since our algorithm needs just the index of one direction at any time, we report such value in "max", whereas "sum" is the size of all our indexes in both directions. The square is the size of the index in [3] built on the concatenation of all the approximately 8600 bacterial genomes in NCBI (34 billion characters): such a string should be moderately repetitive.

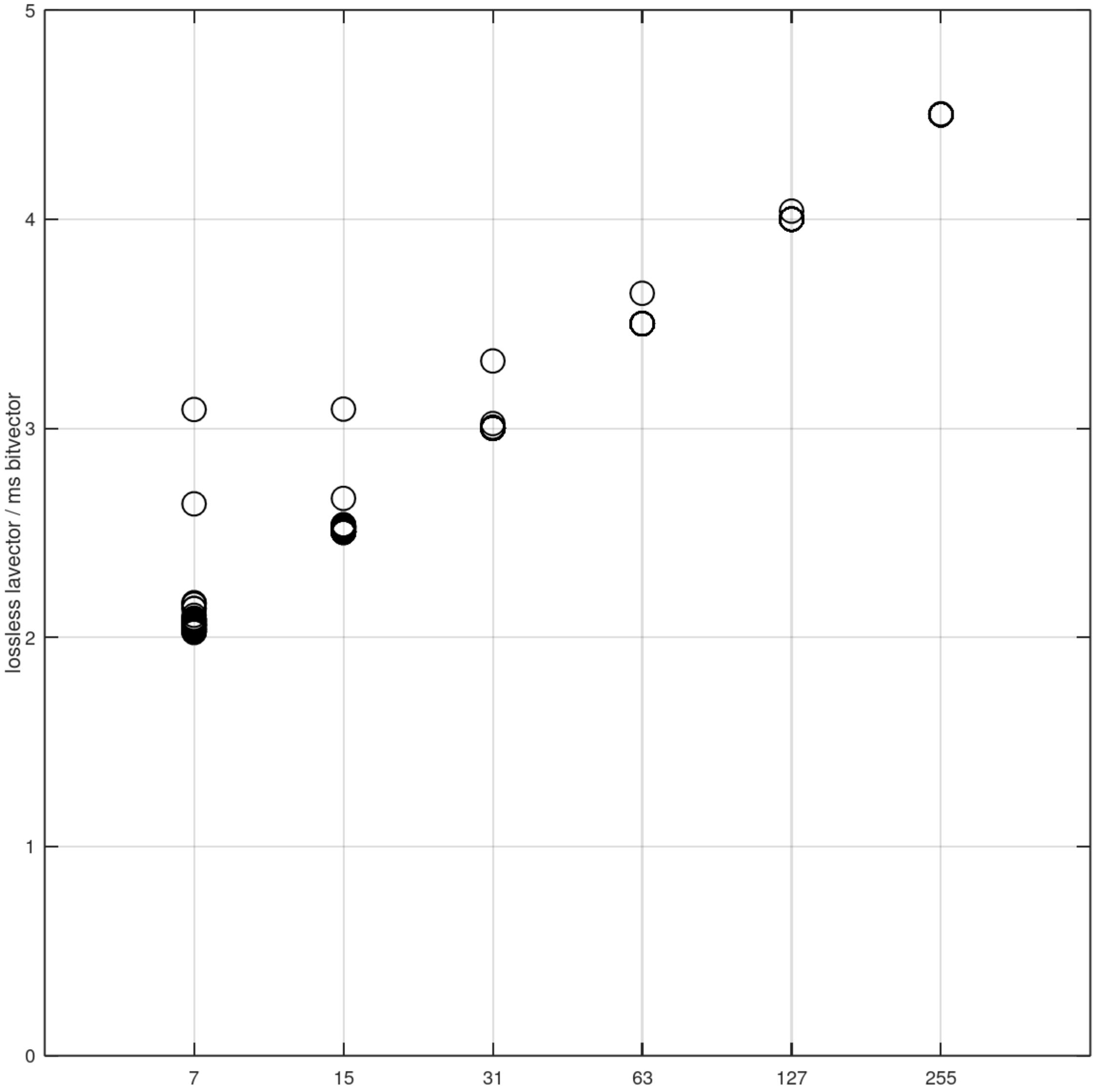


Figure 3: Size of the lossless `la_vector` implementation in [2] applied to the `ms` bitvector of some pairs of large genomes in the ACS dataset, compared to the original size of the `ms` bitvector. Horizontal axis: the maximum error $(2^{c-1} - 1)$ that can be represented by a correction encoded in $c$ bits.

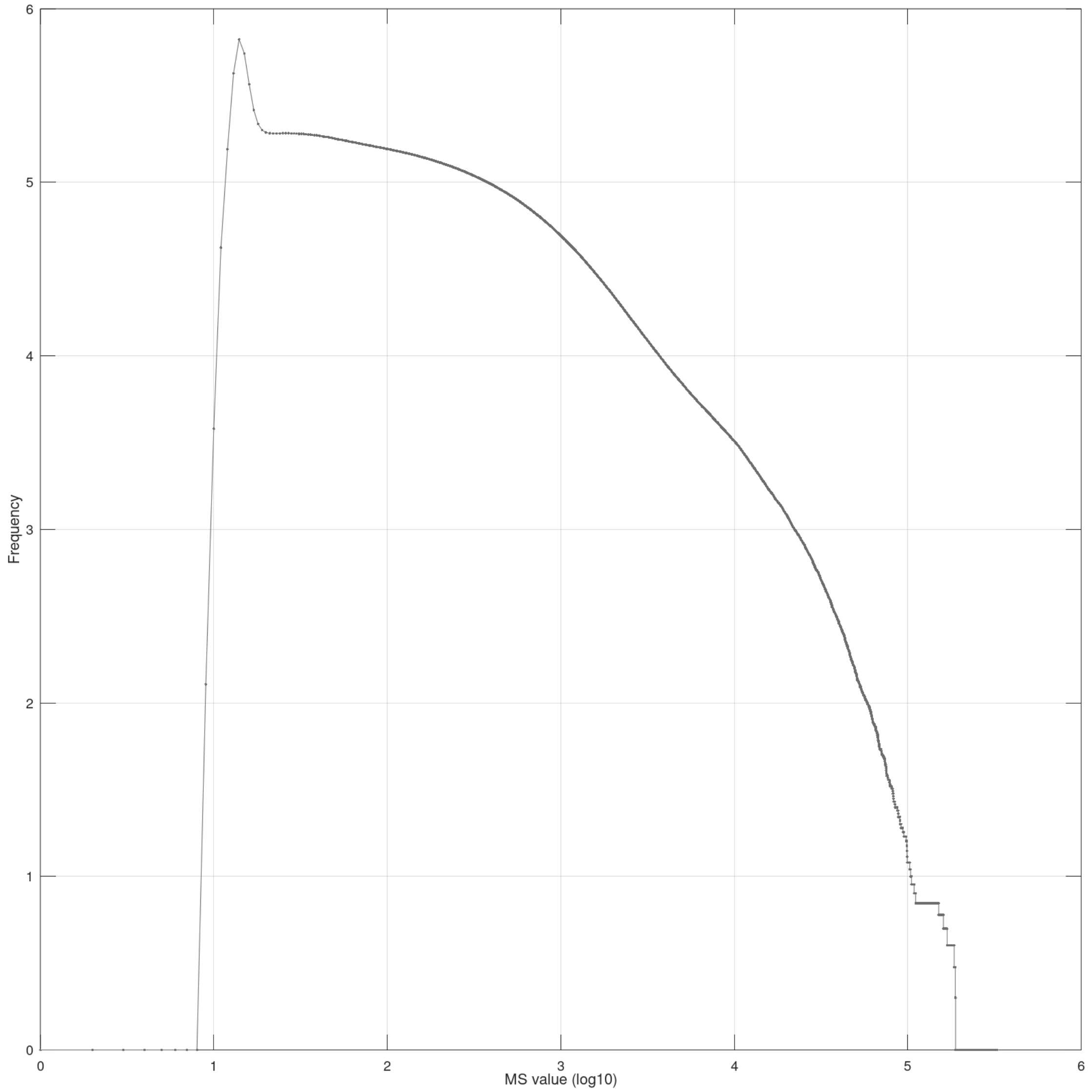


Figure 4: Histogram of MS values between *Homo sapiens* and *Pan troglodytes*. The vertical axis is in logarithmic scale (base 10). The histogram for pairs of unrelated genomes is similar up to the peak, but thereafter it goes quickly to zero instead of having a long tail.

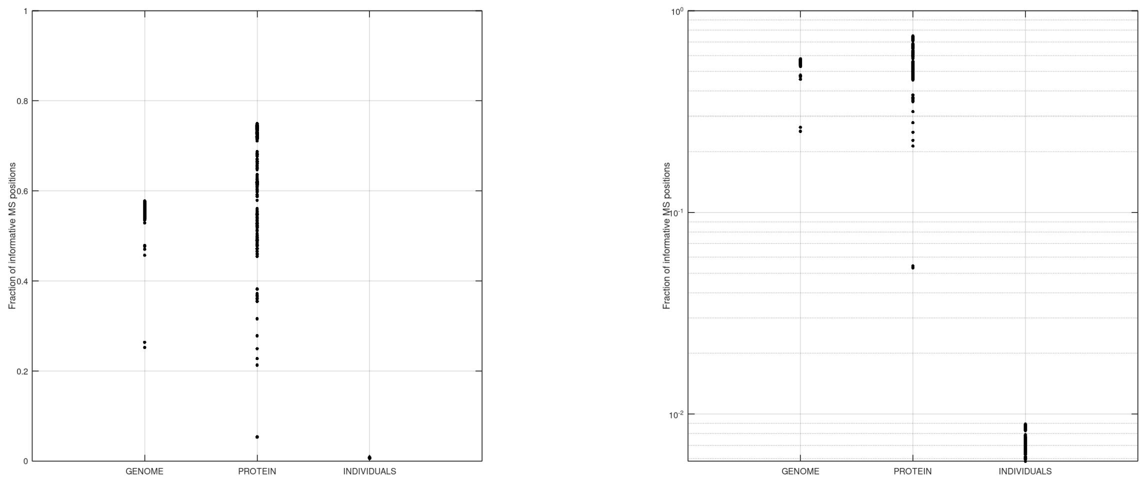


Figure 5: Fraction of *informative positions*, i.e. of positions $i$ such that $\mathsf{MS}[i] > \mathsf{MS}[i-1]-1$, for every pair of sequences in the ACS dataset ("genome" and "protein") and in the dataset of human individuals. The right panel shows the same data as the left one, but in logarithmic scale.

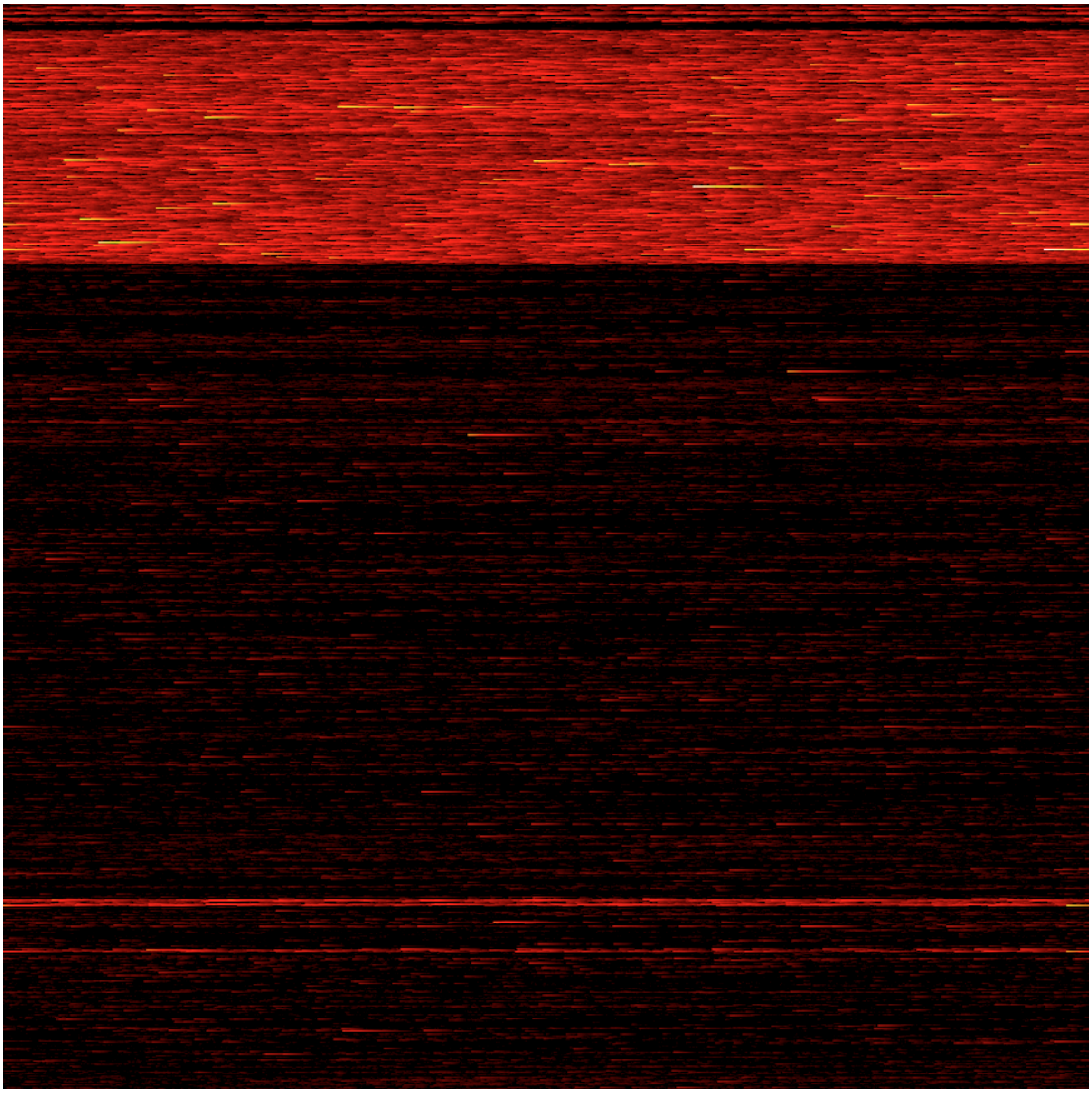

Figure 6: The matching statistics array highlights large-scale patterns of similarity. The figure shows the first hundred million values of the MS array between *Homo sapiens* (query) and *Pan troglodytes* (text). The array is displayed in several rows of fixed length just for visualization purposes. Every pixel is a position in MS. Colors represent MS values (black $<$ red $<$ yellow $<$ white), which range between 5 and 1442.

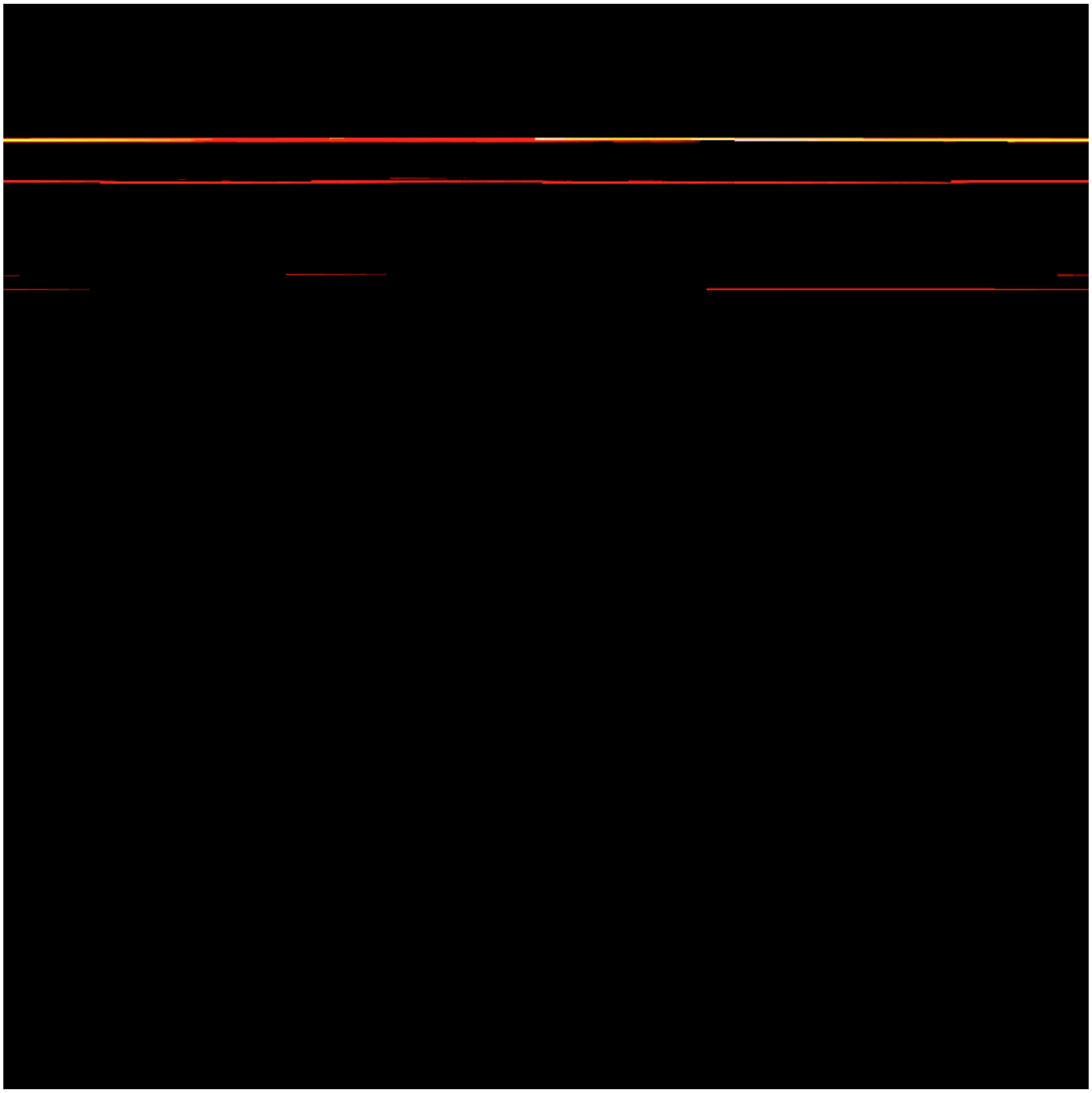

Figure 7: Same as Figure 6, but for *Homo sapiens* (query) and the reverse-complemented of itself (text). MS values range between 5 and 12.4k.

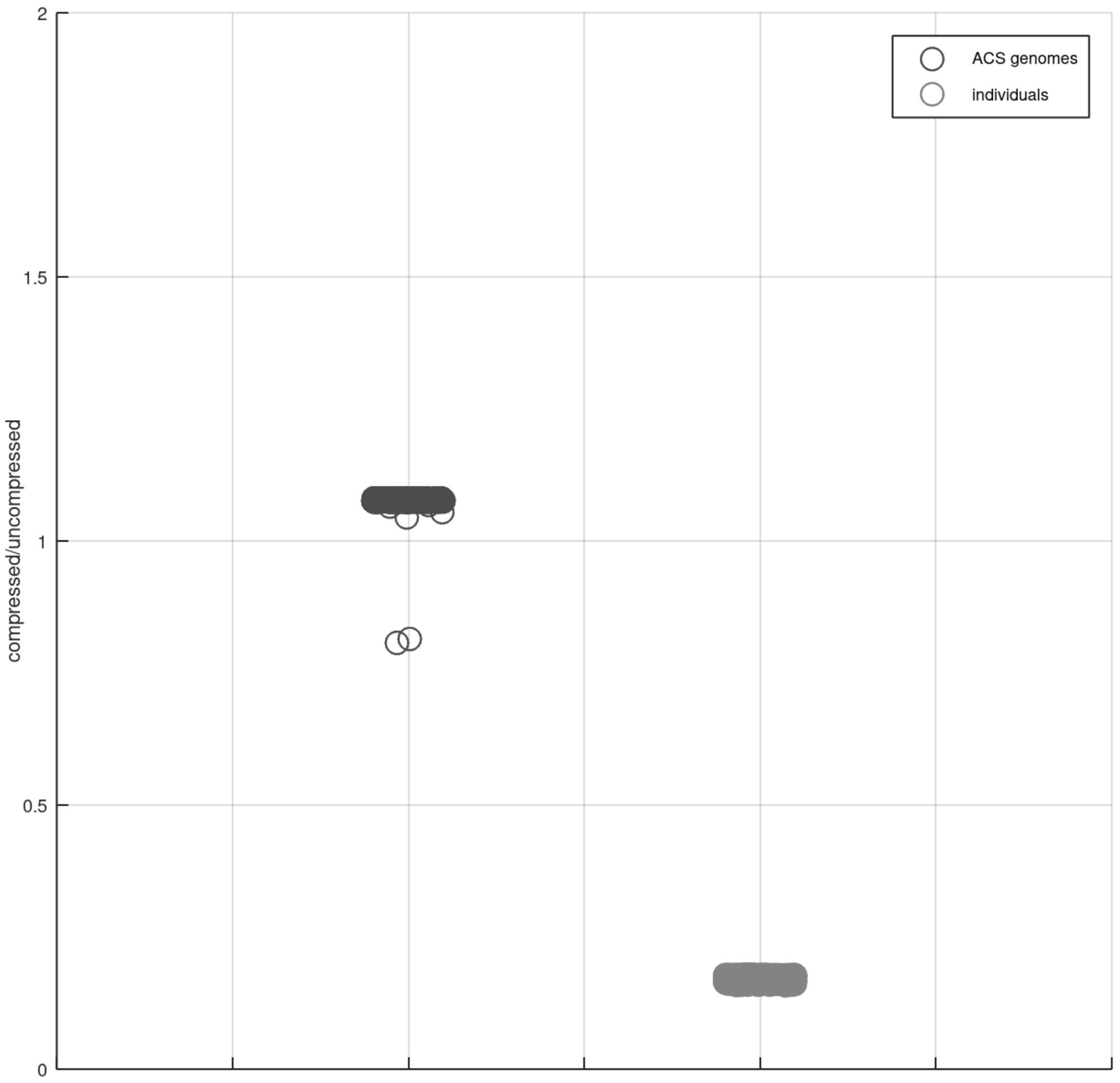


Figure 8: Compressing the `ms` bitvectors of genomes using the `rrr_vector` data structure from the SDSL library [4]: ACS dataset (left) and human individuals dataset (right). Most bitvectors of different species get expanded.

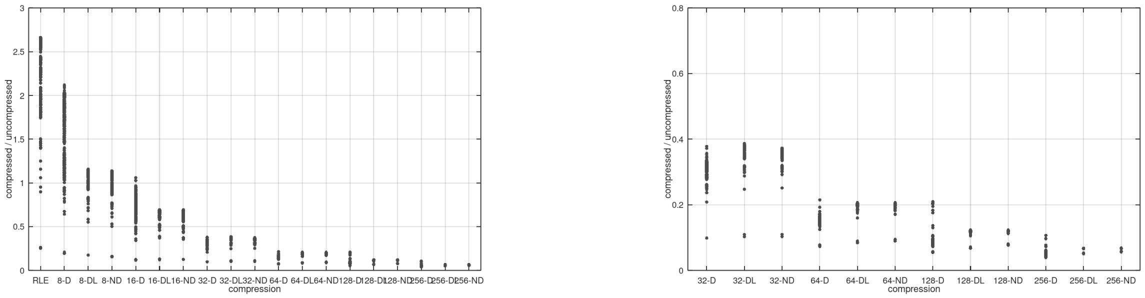


Figure 9: Same as Figure 3 in the paper, but for pairs of proteomes from different species in the ACS dataset. Negative values of MS are not allowed in the lossy variants. The right panel is a zoom-in of the left panel.

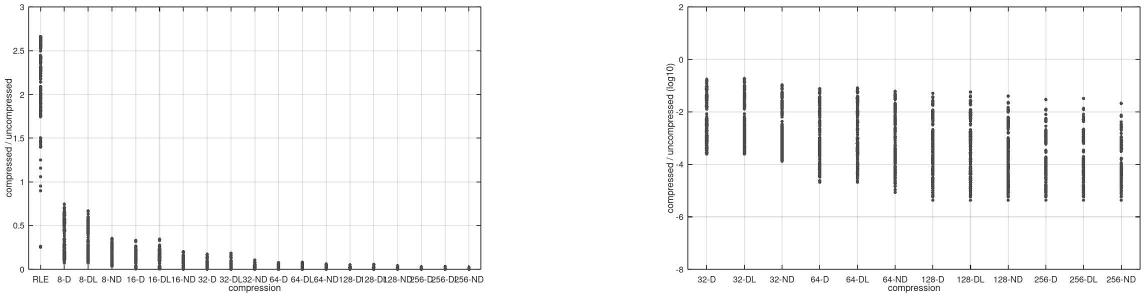


Figure 10: Same as Figure 9, but in this case negative values of MS are allowed.

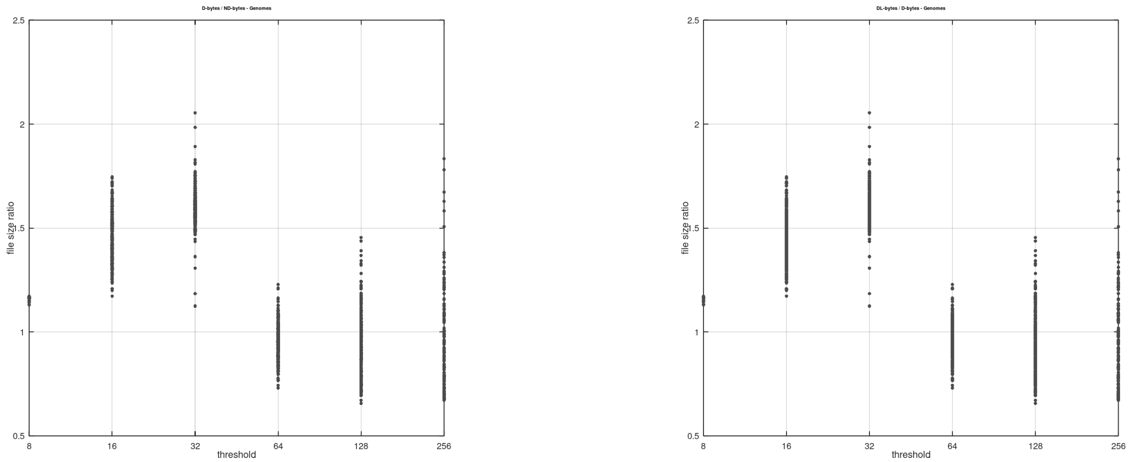


Figure 11: Ratios of D over ND (left panel) and of DL over D (right panel), for every pair of genomes from different species in the ACS dataset. Negative MS values are not allowed. Sizes are measured on disk, and refer to the `RLEVector` data structure by [7].

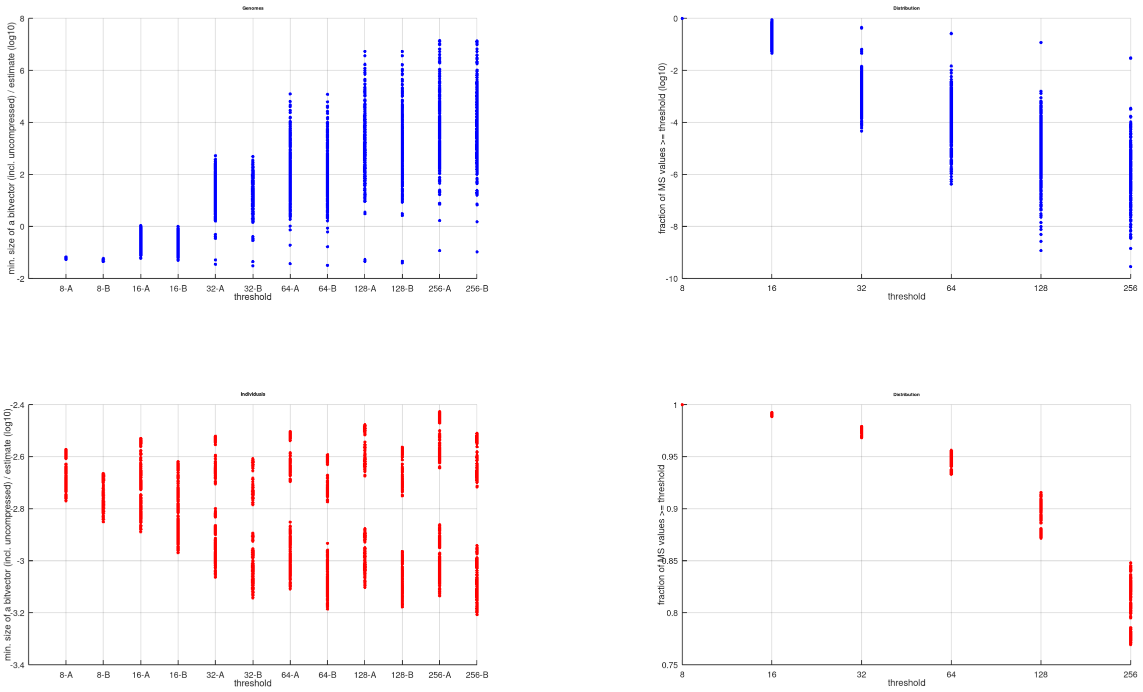


Figure 12: Comparing our lossy schemes to a simple baseline that stores just the MS values above threshold (see Section 4 in the paper for details). Top panels: genomes from different species in the ACS dataset. Bottom panels: genomes from the human individuals dataset. Right panels: fraction of values above threshold. Left panels: ratio between the smallest disk size of one of our bitvectors (i.e. the minimum over D, DL, ND, and not permuted), and the disk size of the $A_\tau$ and $B_\tau$ schemes.

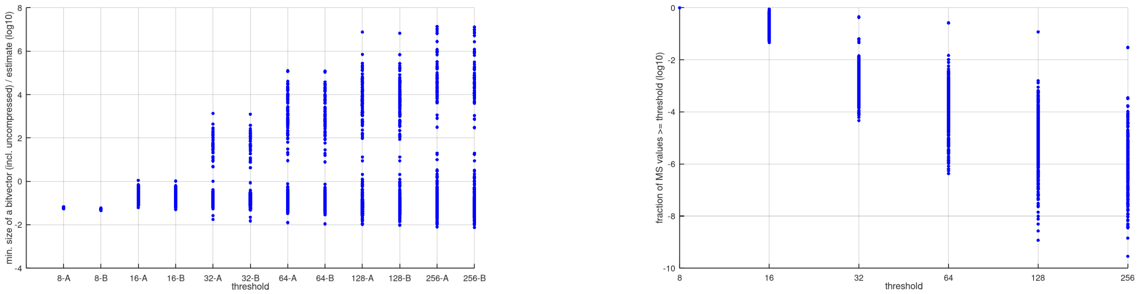


Figure 13: Same as Figure 12 (top), but allowing negative values in the permuted bitvectors.

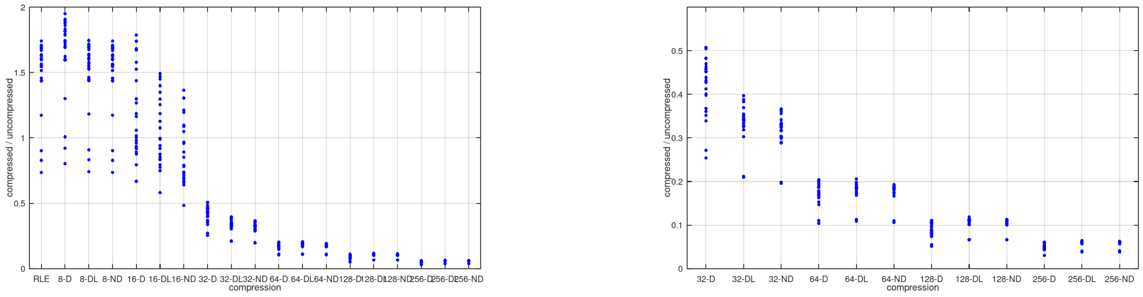


Figure 14: Same as Figure 3 in the paper (left panel), but for the PLCP array of the genomes in the ACS dataset. The right panel is a zoom-in of the left panel.

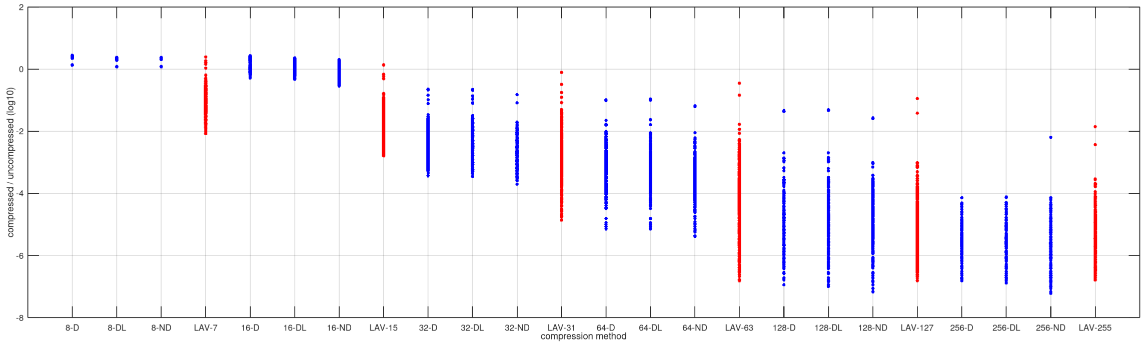


Figure 15: Comparing our lossy schemes to a lossy variant of `la_vector` on the ACS genome dataset. $\tau$-D, $\tau$-DL, $\tau$-ND: disk size of our bitvector permutations with threshold $\tau$, negative values allowed, and encoded with the `RLEVector` data structure by [7] (blue). LAV-$\epsilon$: disk size of a lossy variant of the `la_vector` implementation [2] in which correction values are not stored (red). $\epsilon$ is the maximum error $(2^{c-1}-1)$ that can be introduced when discarding the corrections (encoded in $c$ bits).

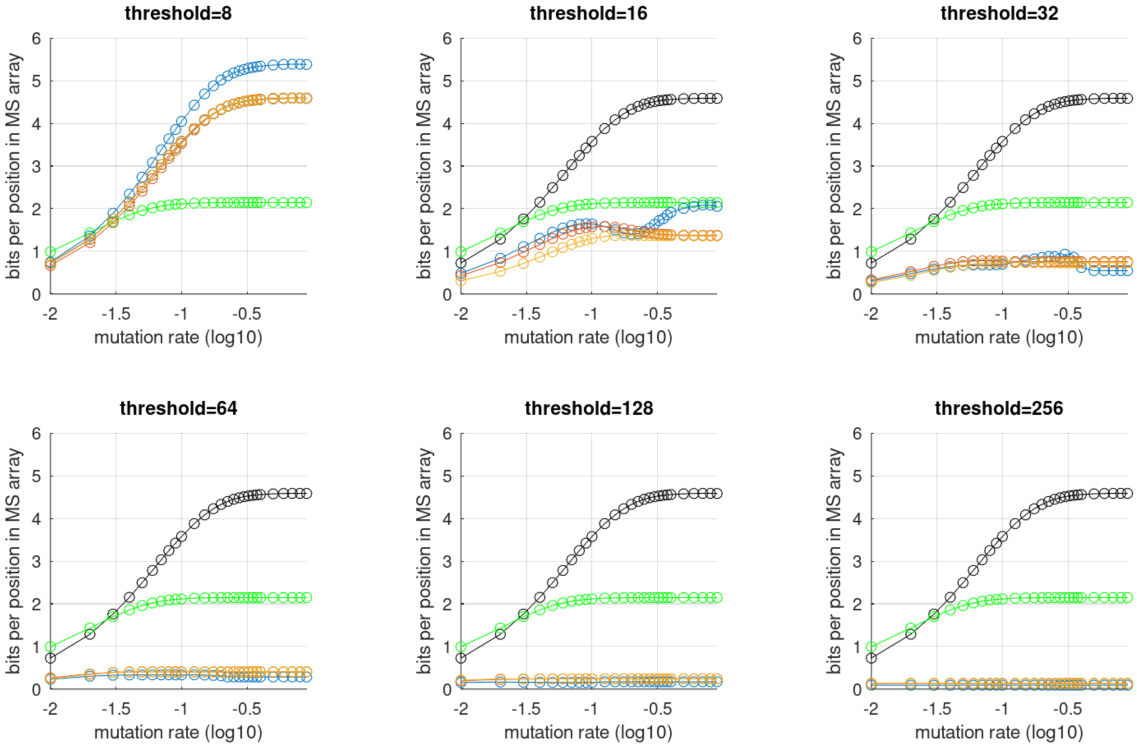


Figure 16: Efficiency of our lossy compression schemes with varying mutation rate between query and text. Text: human chromosome 1. Query: a 10 Mbps prefix of the text, with added mismatches at variable rate (horizontal axis). Green: disk size of the `rrr_vector` data structure from the SDSL library [4] built on the original `ms` bitvector. Black: disk size of the `RLEVector` data structure by [7] built on the original `ms` bitvector. Other colors: disk size of `RLEVector` built on the D (blue), DL (red) and ND (yellow) permutations. Negative values of MS are not allowed. The uncompressed `ms` bitvector takes 2 bits per position, so plain RLE *expands* the bitvector for mutation rates $\geq 0.04$.

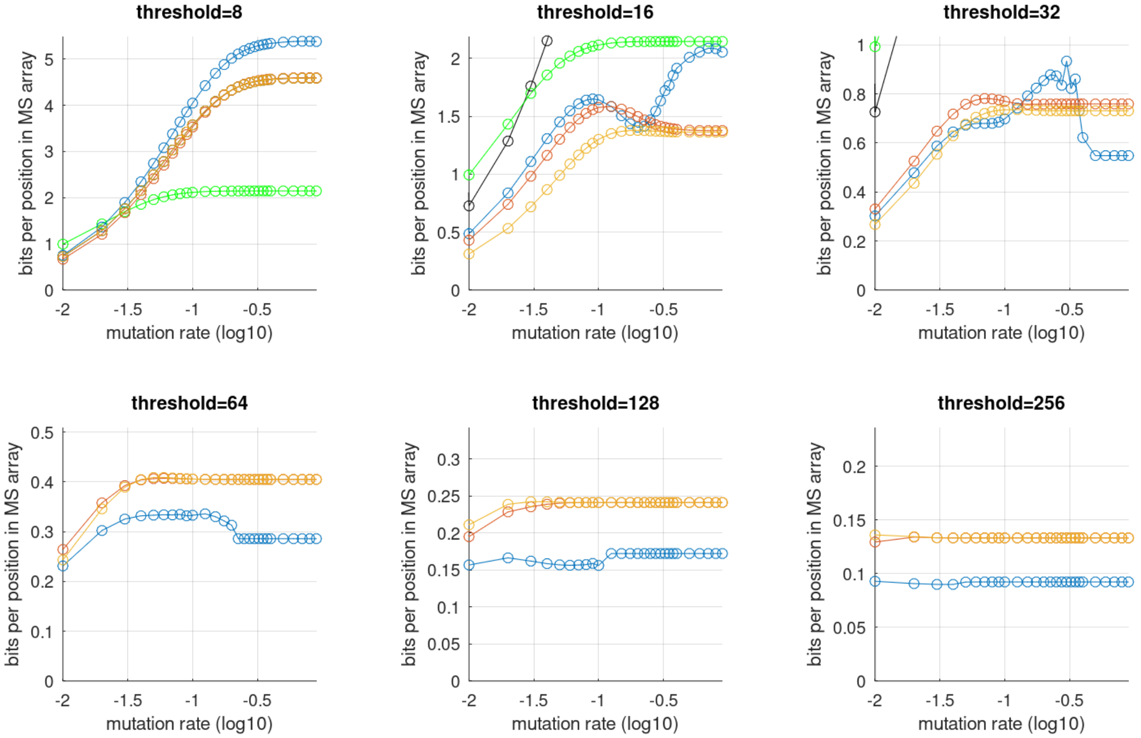


Figure 17: Zoom of Figure 16. At high mutation rates (dissimilar sequences), DL and ND (red and yellow) are almost equivalent, and D (blue) becomes useful only for high values of the threshold. At low mutation rates (similar sequences), ND is equivalent to DL or preferable to it for all values of the threshold.

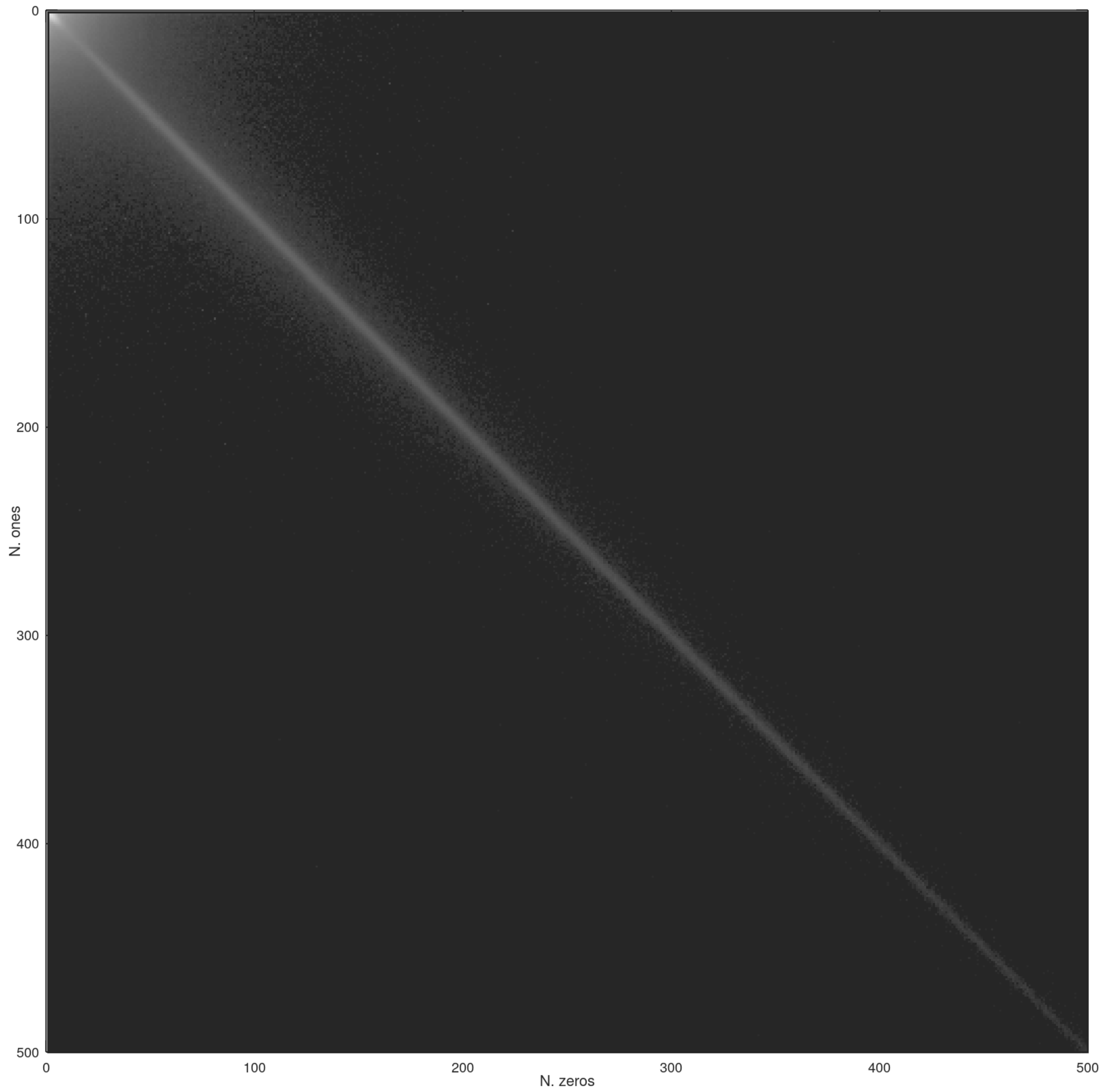


Figure 18: Correlation between the length of a run of zeros and the length of the following run of ones in the `ms` bitvector of *Homo sapiens* and *Pan troglodytes*. Every pair of lengths is a cell of the matrix; the value in a cell is the logarithm of the number of observed pairs (black means low, white means high). We detected no correlation between the length of a run of zeros and the length of the *preceding* run of ones (or the length of any of the preceding runs of zeros). A similar trend appears in the bitvector encoding of the PLCP array of the human genome (data not shown) [6].

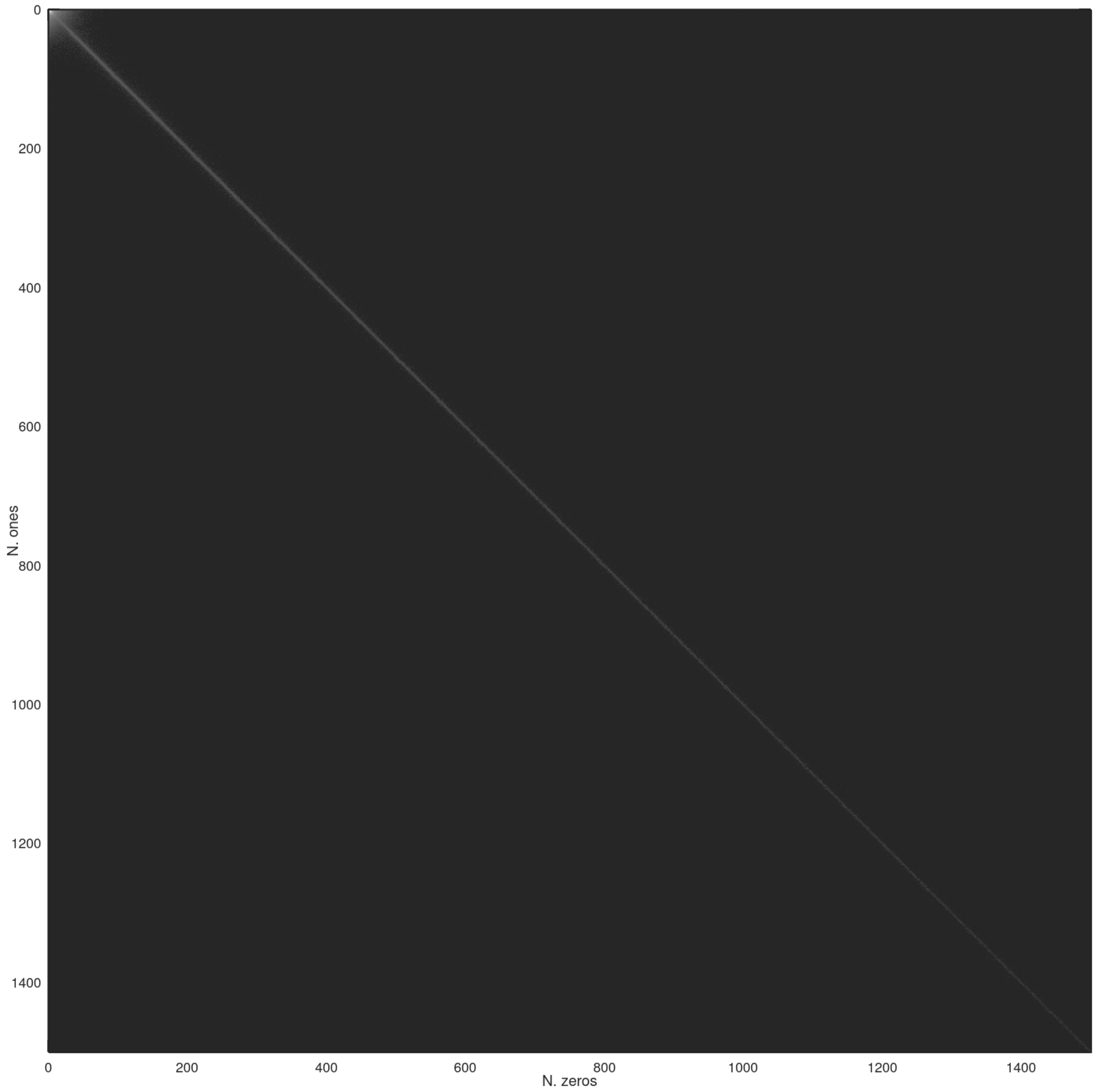


Figure 19: Same as Figure 18, but for a pair of chromosome 1 sequences from human individuals.

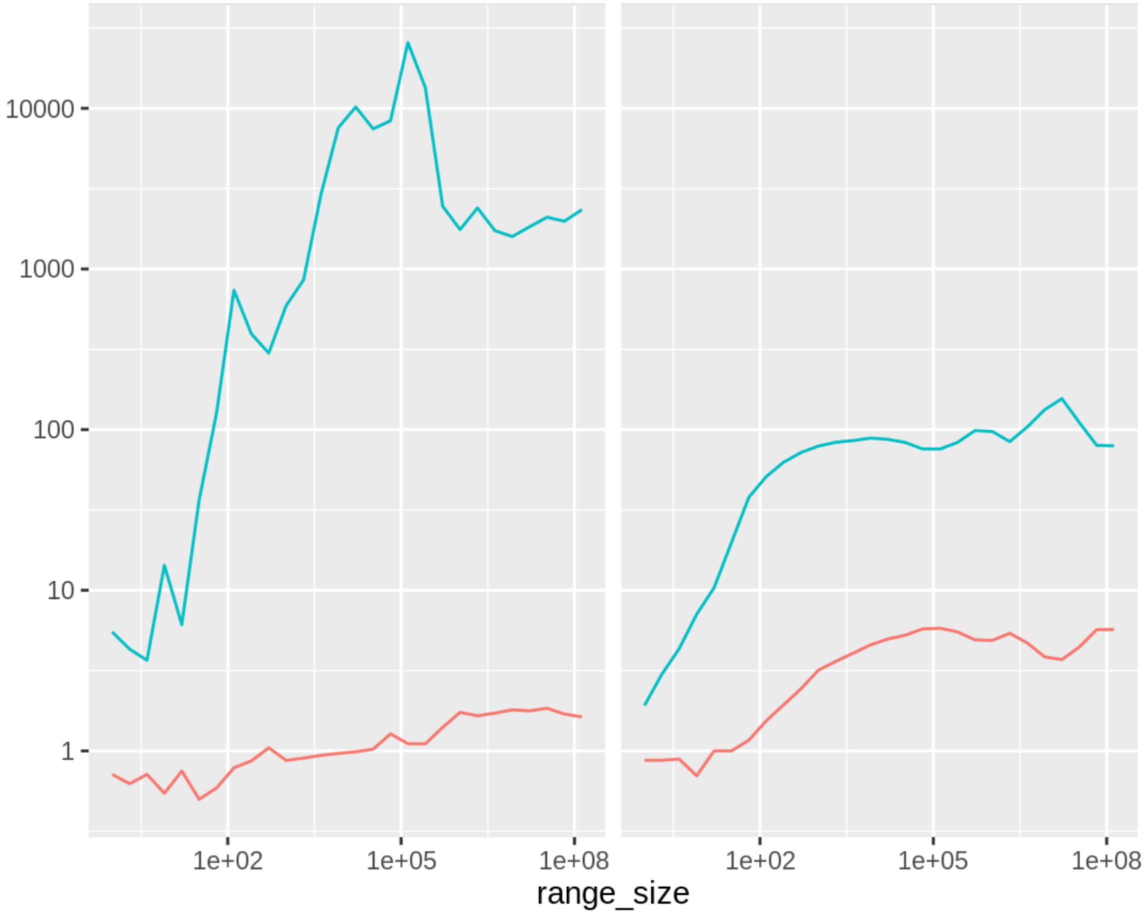


Figure 20: Scanning the `ms` bitvector in range-max queries: ratio between the time of the baseline approach that accesses every bit of the bitvector, and the time of our optimized code. Red: `bit_vector` data structure from SDSL [4]. Green: `RLEVector` data structure from [7]. Left: chromosome 1 of two human individuals. Right: *Homo sapiens* and *Mus musculus*. Every point is the average of 50 random queries of the same size.

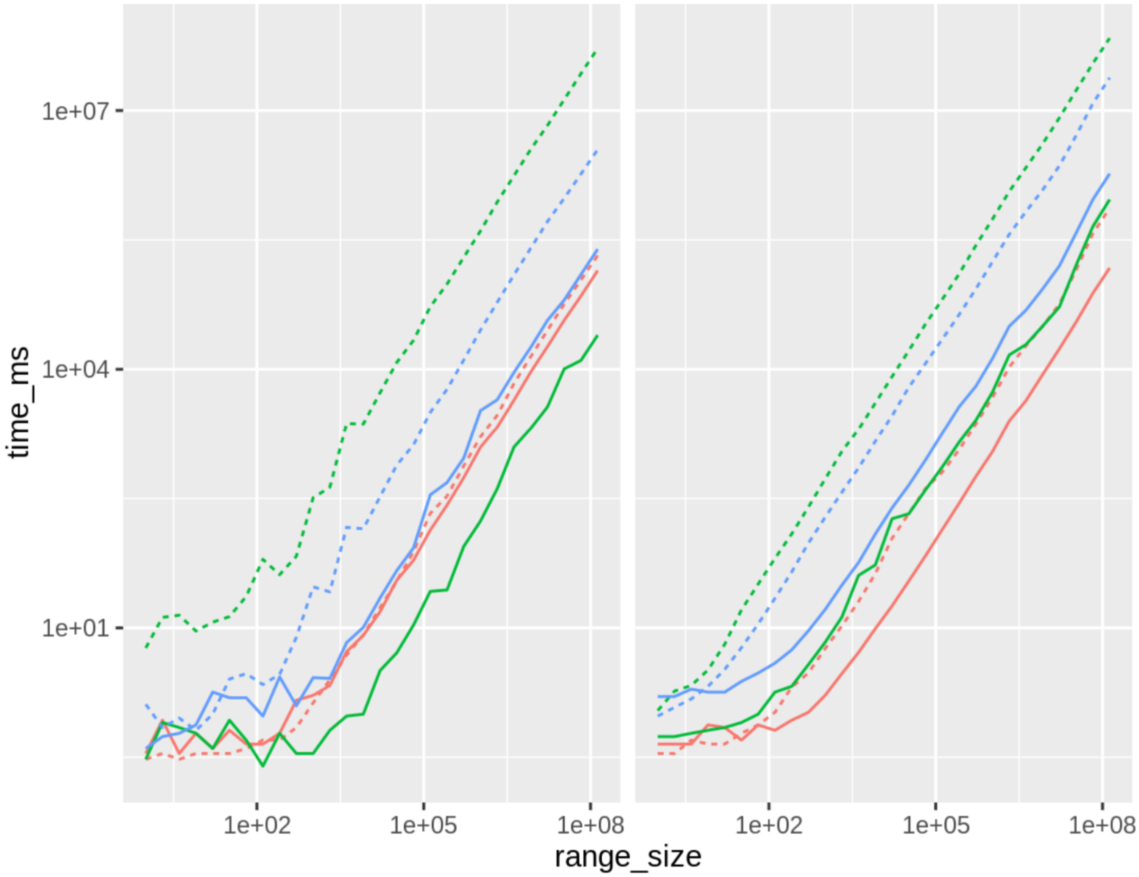


Figure 21: Scanning the `ms` bitvector in range-sum queries: time of the baseline approach that accesses every bit of the bitvector (dotted lines), and time of our optimized code (continuous lines). Red: `bit_vector` data structure from SDSL [4]. Green: `RLEVector` data structure from [7]. Blue: `rrr_vector` from SDSL. Left: chromosome 1 of two human individuals. Right: *Homo sapiens* and *Mus musculus*. Every point is the average of 50 random queries of the same size.

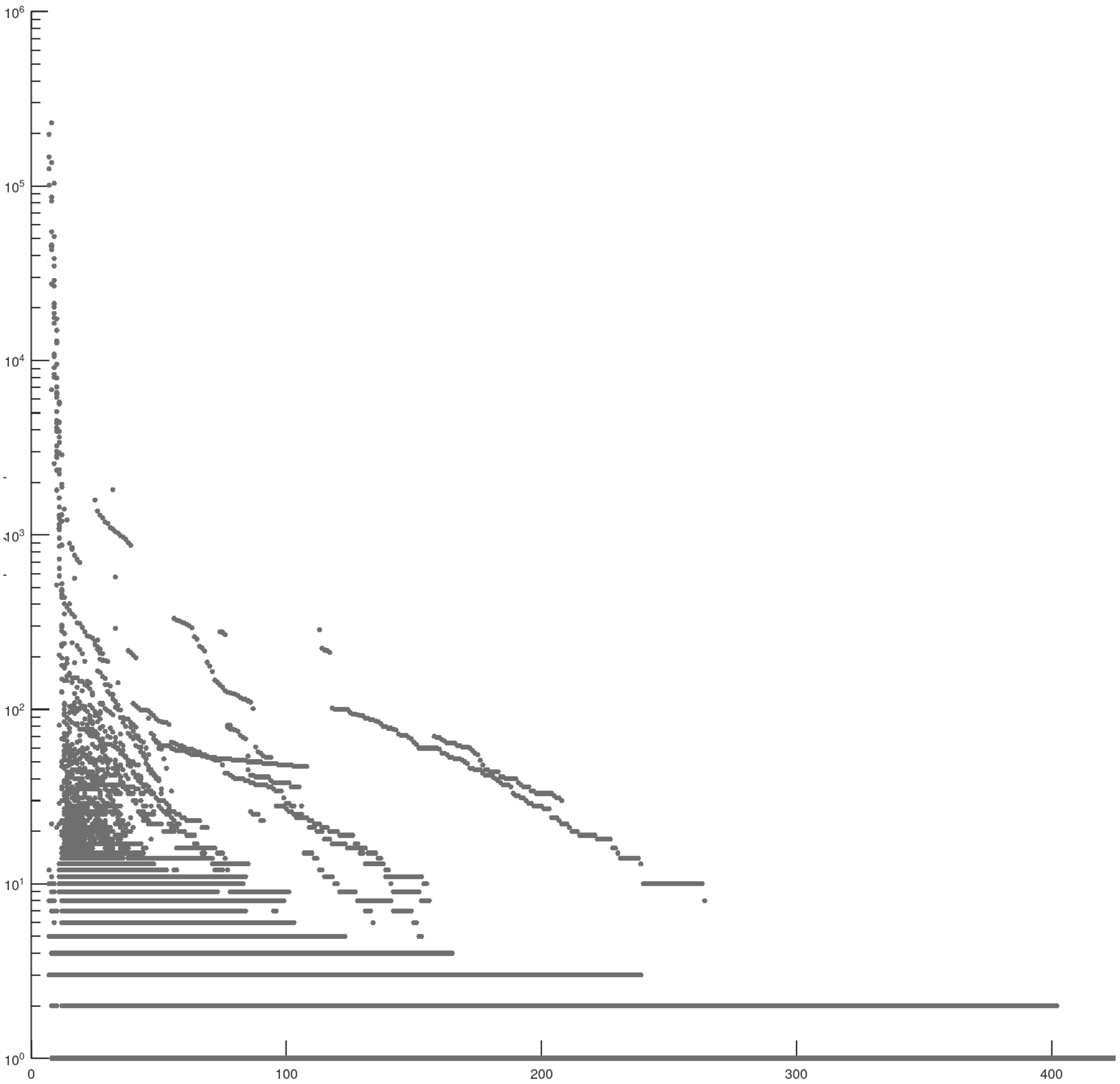


Figure 22: Long matching statistics values between the query (*Homo sapiens*) and the text (*Pan troglodytes*) can have high frequency in the text. Horizontal axis: matching statistic value. Vertical axis: frequency in the text (logarithmic scale). This suggests that the two genomes share exact repeats.

## References

[1] Djamal Belazzougui, Fabio Cunial, and Olgert Denas. Fast matching statistics in small space. In *Proceedings of the 17th International Symposium on Experimental Algorithms (SEA 2018)*, volume 103. Schloss Dagstuhl-Leibniz-Zentrum fuer Informatik, 2018.

[2] Antonio Boffa, Paolo Ferragina, and Giorgio Vinciguerra. A "learned" approach to quicken and compress rank/select dictionaries. In *2021 Proceedings of the Workshop on Algorithm Engineering and Experiments (ALENEX)*, pp. 46–59. SIAM, 2021.

[3] Christina Boucher, Travis Gagie, I Tomohiro, Dominik Köppl, Ben Langmead, Giovanni Manzini, Gonzalo Navarro, Alejandro Pacheco, and Massimiliano Rossi. PHONI: Streamed matching statistics with multi-genome references. In *2021 Data Compression Conference (DCC)*, pp. 193–202. IEEE, 2021.

[4] Simon Gog, Timo Beller, Alistair Moffat, and Matthias Petri. From theory to practice: plug and play with succinct data structures. In *13th International Symposium on Experimental Algorithms (SEA 2014)*, pp. 326–337, 2014.

[5] Sven Rahmann. Fast and sensitive probe selection for dna chips using jumps in matching statistics. In *Computational Systems Bioinformatics. CSB2003. Proceedings of the 2003 IEEE Bioinformatics Conference. CSB2003*, pp. 57–64. IEEE, 2003.

[6] Kunihiko Sadakane. Compressed suffix trees with full functionality. *Theory of Computing Systems*, 41(4):589–607, 2007.

[7] Jouni Sirén. Compressed suffix arrays for massive data. In *International Symposium on String Processing and Information Retrieval*, pp. 63–74. Springer, 2009.

[8] Igor Ulitsky, David Burstein, Tamir Tuller, and Benny Chor. The average common substring approach to phylogenomic reconstruction. *Journal of Computational Biology*, 13(2):336–350, 2006.
